# Cases Illustrative of the Pathology and Surgical Treatment of Some of the Diseases of the Testicle; with Observations

**Published:** 1826-10

**Authors:** B. C. Brodie


THE LONDON
### Medical and Physical Journal.

No. 332, VOL. LVI.] OCTOBER, 1826. [No. 4, *New Series*.

For many fortunate discoveries in medicine, and for the detection of numerous errors, the world is indebted to the rapid circulation of Monthly Journals; and there never existed any work, to which the Faculty, in Europe and America, were under deeper obligations, than to the Medical and Physical Journal of London, now forming a long, but an invaluable, series.—RUSH.

### ORIGINAL PAPERS,
AND
### CASES OBTAINED FROM PUBLIC INSTITUTIONS AND OTHER AUTHENTIC SOURCES.

### DISEASES OF THE TESTICLE.

*Cases illustrative of the Pathology and Surgical Treatment of some of the Diseases of the Testicle; with Observations.* By B. C. Brodie, F.R.S. &c. &c.

#### I. *Wasting of the Testicle.*

Where the organisation of the testicle has been destroyed by inflammation and its consequences, the remains of that organ, in some instances, become gradually reduced to a very small size. Here the wasting of the testicle is merely the effect of another disease; but there are other cases in which a wasting of the testicle takes place as a primary affection.

Case I. *Dissection of a Wasted Testicle, where there were no marks of other disease.*

In a middle-aged man, who died in St. George's Hospital, of tubercles in the lungs, I found the left testicle of the usual size, and in all respects of a natural appearance. The right testicle was wasted, so that it was of not more than one-sixth or one-eighth of the ordinary size. The two layers of the tunica vaginalis adhered universally to each other. On examining the structure of the testicle itself, the tubuli testis, or rather the remains of them, were still perceptible; but they did not admit of being drawn out into threads as usual. The tortuous tube of the epididymis was also distinct, but much diminished in diameter. The epididymis, however, was not wasted in the same degree as the rest of the testicle. The vas deferens was pervious throughout its whole extent. There was no thickening or induration of any of the parts connected with the testicle, nor any marks of former inflammation, except we were to regard as such the adhesion of the two layers of the tunica vaginalis to each other.*

* The testicle is preserved in Mr. Brodie's collection of preparations of morbid anatomy.

Case II. *Wasting of the Testicle, induced by Onanism.*

George P—, a stout young man, twenty years of age, admitted into St. George's Hospital, June 12th, 1805. He complained of pain in the left testicle, which was wasted to about one-third of its natural size, soft and flaccid to the touch, wholly unlike the other.

He said that he had never received a blow, nor had he laboured under gonorrhœal inflammation of the testicle; but that, for five years previous to his admission, he had been addicted to onanism. He had carried this practice to such an extent, that, during the above-mentioned period, he had seldom passed a day without repeating it, at the least, once. Ten months before his admission, he first experienced a severe pain in the left testicle; after this the testicle enlarged in size. The enlargement was soon followed by a perceptible diminution, and from this time the testicle continued to waste. During the whole course of the disease, it had been attended with severe pains in the testicle, and very great depression of spirits. The latter was strongly depicted in the countenance, giving it a peculiarly melancholy and gloomy appearance.

He was directed by the surgeon, under whose care he was, to take the Sulphate of Iron, with Tincture of Cantharides, internally; and blisters were applied to the scrotum.

July 6th.—There was no increase in the size of the wasted testicle; but he said that the pain was diminished, and he now referred it to the groin, rather than to the testicle itself.

July 10th.—He complained of pain in the right testicle also; and he was directed to apply a blister over it.

Soon afterwards, he was made an out-patient.

August 13th.—The pains in both testicles had entirely ceased: the size of the wasted testicle remained unaltered.

September 9th.—He remained in the same state. I did not see him afterwards.

Case III. *Wasting of the Testicle, induced by too much indulgence in sexual intercourse.*

In September 1820, I was consulted by a patient, thirty-one years of age, under the following circumstances:—Both testicles were wasted, and reduced to so small a size as to be only just perceptible through the integuments of the scrotum. He sometimes experienced a slight degree of sexual desire, but never sufficient for him actually to be connected with the other sex, and there was never any seminal discharge. He said that he had begun having intercourse with women at the early age of fourteen years; that he had indulged himself to excess while yet a boy, and had continued to do so for several years. When about twenty years of age, he laboured under inflammation of both testicles; which arose from some accidental cause, and was removed by the usual means. Some time afterwards, (but, as far as he could recollect, not immediately,) the testicles began to waste, and his sexual desires to decline; and, in the course of two or three years, he became what he was when I saw him. I observed that he had very little beard.

### II. *Varicose Veins of the Testicle.*

I have no doubt that cases have occurred in which the veins of the testicle have been varicose to so great an extent, and the consequent sufferings of the patient have been so severe, as to render it advisable for him to submit to the operation of castration. I have not, however, myself met with a case in which I felt justified in proposing such a proceeding to my patient; and the following is the only instance in which I have attempted to give relief by an operation on the veins themselves.

Case IV. *Varicocele, relieved by the division of the varicose vessels.*

James Adams, twenty-one years of age, admitted into St. George's Hospital, April 2d, 1817; having been recommended to my care by Mr. Andrews, of Watford, now of Arlington-street. He had varicose veins of the left spermatic chord and testicle, which were principally situated at the posterior part of the epididymis, forming a cluster in this situation. The varicose tumor was not very large, but he suffered at times a very considerable degree of pain, especially in the evening, when the veins were more distended than in the morning.

He was directed to be cupped in the loins; to take moderate doses of Sulphate of Magnesia, so as to keep the bowels gently open; and to apply a cold lotion to the groin and testicle.

Under this treatment no improvement took place. On further examination, I found that the pain was referred almost wholly to the cluster of varicose veins situated at the posterior part of the epididymis; and I was induced to believe that the sufferings of the patient arose from the pressure of the tumor on some contiguous nerve or nerves, and that, if the dilated veins forming it could be obliterated, the pain would be relieved. With this impression on my mind, on the 14th of April, I performed the following operation:

I divided, with a sharp-pointed bistoury, the skin and cellular texture at the posterior part of the scrotum, so as to expose the varicose cluster; and then, by a second incision, I divided the varicose cluster itself, cutting through its centre. When first exposed, the cluster was of about the size of a horse-bean, of a purple colour: on being divided, it immediately collapsed, and there was a slight venous hemorrhage. Some cold lotion was applied, the wound being allowed to remain open, in order to favour the escape of the blood, and prevent its effusion into the cellular texture of the scrotum.

Some inflammation and tumefaction of the scrotum followed the operation; but there was no fever, nor much uneasiness of any kind.

April 21st.—The wound was healing, and the patient was entirely free from the pain which he had complained of before the operation.

May 13th.—The wound was healed: he was free from pain. A slight degree of hardness remained where the divided cluster of veins was situated. Discharged, cured.

### III. *Chronic Inflammation of the Testicle, yielding to the use of mercury.*

It is well known to surgeons that there is one species of chronic inflammation of the testicle, which is very little under the dominion of those remedies which are useful in common inflammatory affections, but which yields readily to the use of mercury. The five following cases are intended to illustrate the history of this disease, when the progress of it is arrested at an early period, that is, previous to the formation of abscess or ulcer, and while the natural structure of the testicle is not materially impaired.

Case V. *Chronic Inflammation of both Testicles, occurring in combination with Disease of the Urethra, and yielding to the use of mercury.*

A. B—, thirty-eight years of age, had laboured for some years under a stricture of the urethra, attended with an unusual degree of irritation, for which he was in the habit of introducing bougies with his own hand. In the summer of 1815, he consulted me on account of an affection of both testicles. They were enlarged to about double their natural size, hard to the touch, knobbed and irregular on the surface. There was pain and tenderness, but not sufficient to prevent his going about his usual occupations, and there was no fever. He said that, a fortnight before, he had been seized with pain in the loins, extending, in the course of the spermatic chord, to the left testicle; and that then this testicle began to be enlarged. A few days afterwards, he experienced pain in the course of the spermatic chord of the other side, and this was followed by an enlargement of the right testicle.

Leeches and cold lotions were applied; the application of leeches was subsequently repeated.

Finding that some fluid was collected within the tunica vaginalis of one side, I made a puncture with a lancet, and about half an ounce of serum was discharged. No amendment took place under this treatment, and fluid became again collected within the tunica vaginalis. I then prescribed the Oxymuriate of Mercury, to be taken in the form of pills. As soon as the mercury acted slightly on the system, the enlargement of the testicles began to subside; the fluid in the tunica vaginalis disappeared; and, in about a month after the commencement of the mercurial course, he was quite recovered, the testicles being restored to their natural condition.

I saw this patient two years afterwards, and he had continued well.

Case VI. *Chronic Inflammation of the Testicle, occurring in combination with rheumatic pains and other cachectic symptoms, and yielding to the use of Mercury and Sarsaparilla.*

A. B—, twenty-nine years of age, consulted me on the 15th of May, 1816. At this time the left testicle was slightly enlarged and indurated, and there was a small quantity of fluid in the tunica vaginalis. The right testicle was enlarged to twelve or fifteen times its natural size. This testicle was hard, somewhat painful, and tender. He complained of pain in the loins and pains in the limbs; there was an ulcer on one thigh, and another on one arm; and there were a few red-coloured eruptions on some parts of his body.

He said that, since the year 1809, he had been subject to pains in the limbs, resembling those of rheumatism: these pains occurred chiefly in the winter, and sometimes disabled him for six successive months. Occasionally he had cough and pain of the chest; and, in 1814, he had glandular swellings in the neck, and an abscess burst in the throat. After this, the pains in the limbs were, on the whole, less severe. The eruptions on the skin first appeared in the year 1812. About Christmas 1815, both testicles became swollen, with severe pain in them and in the loins. The swelling was rapid, attaining a large size in a few hours. In about three weeks, the pain in great measure subsided, but the swelling of the testicles remained.

During these years of ill health, he lost flesh, so that, although in the year 1806 he weighed thirteen stone, his weight was reduced to nine stone and a half, at the time of my being consulted.

I prescribed the following mixture:—℞. Decocti Sarsaparillæ Oj.; Extr. Sarsaparillæ ʒij.; Hydrargyri Oxymuriatis gr. ¼, fiat mistura, cujus sumat partem tertiam ter quotidie. I also directed the application of some leeches to the right testicle.

May 26th.—The fluid was almost entirely absorbed from the left tunica vaginalis. The right testicle was perceptibly diminished in size. The pains in the limbs were relieved.

June 19th.—The right testicle was not more than one-sixth of its former size; it was much softer, and free from pain. Very little fluid remained in the tunica vaginalis of the left testicle. He had no pains in the limbs, nor any remains of the eruption; the ulcers of the arm and thigh were healed; his appetite was much improved. He slept well, was stronger, and had gained flesh.

The sarsaparilla and oxymuriate of mercury were continued for some weeks.

In October, he was free from all complaint.

November 18th, 1817, he continued in perfect health. The right testicle was, in all respects, in a natural state. Occasionally there was a small quantity of fluid in the left tunica vaginalis.

Case VII. *Chronic Inflammation of the Testicle, yielding to the use of Mercury, and occurring in a person who had no other disease.*

John Denham, thirty-four years of age, admitted into St. George's Hospital, May 9th, 1815. He said that, five years ago, he had a painful swelling of the left testicle, attended with pain in the loins and lower part of the abdomen. Three weeks after the swelling was first observed, it was punctured by a surgeon, and some fluid was evacuated. According to the patient's own report, he was well afterwards, and continued so until about two months before he came to the hospital, when the same testicle became again swollen; the swelling being accompanied with pain in the loins, in the lower part of the abdomen, and in the testicle itself. In the course of a month, the pain, in great measure, subsided, but the testicle remained enlarged and tender.

At the time of his admission into the hospital, the left testicle was enlarged to four or five times its natural size, of an oval form, but somewhat prominent at the anterior and inferior part; hard behind, elastic anteriorly; it was also a little tender. He had pain in the loins, but none in the testicle, except on pressure. The right testicle was also enlarged, but much less so than the left. He did not complain that it was painful, even when pressed, and he himself had not discovered that there was any disease in it. The form of the right corresponded to that of the left testicle, being not regularly oval, but prominent at the anterior and inferior part.

May 10th.—Leeches were applied to the left testicle, and he was directed to take ʒss. of the Pilula Hydrargyri daily.

May 11th.—A puncture was made in the tunica vaginalis of the left testicle, and three ounces of serum were evacuated. The indurated testicle was now more distinctly felt, the elastic part of the tumor having been formed entirely by the fluid in the tunica vaginalis. He continued to take the Pilula Hydrargyri in such doses as were necessary to keep his gums moderately sore.

June 3d.—The left testicle was very much diminished in size, and much softer. After the puncture, fluid had again become collected in the tunica vaginalis, but it was now entirely absorbed.

He continued to take the Pilula Hydrargyri, using no other remedies until July 18th, when he went into the country. At this time the left testicle was scarcely larger than natural, and free from all induration. The right testicle was still somewhat hard and enlarged, but less so than formerly.

Case VIII. *Chronic Inflammation of the Testicle connected with Rheumatism, and yielding to the use of Mercury.*

Henry Dodge, twenty-eight years of age, admitted into St. George's Hospital, on the 17th of May, 1826. Three weeks before his admission, he became affected with pain referred to the right groin and loins; in consequence of which, he was compelled, after three or four days, to give up his occupations as a smith. He then observed the right testicle to become swollen and indurated. There was no fever attending the inflammation, which seemed to have a chronic character, and, although it interfered with his pursuing his usual labours, it did not prevent him from walking slowly to a considerable distance. He had been subject to rheumatism, and had been affected with this disease just before the inflammation began in the testicle.

At the time of his presenting himself at the hospital, the right testicle was enlarged, and in some parts, but not universally, indurated. The spermatic chord was also enlarged and indurated, as far as it could be traced in the groin. The testicle was tender, and the patient complained of pain in the loins and groin. He was at this time free from any rheumatic affection.

Leeches were applied to the testicle, and on the 17th of May the following pill was directed to be taken every morning and evening:—℞. Calomelanos gr. ij.; Pulv. Antimonialis gr. j.; Opii pulv. gr. ss. fiat pilula.

June 6th.—The gums being very sore, the pill formerly prescribed was changed for five grains of the Pilula Hydrargyri, to be taken twice daily. The testicle was much reduced in size.

10th.—The gums were less sore. The bowels were too much relaxed, probably from the use of the mercury.

Some Chalk Mixture and Aromatic Confection were prescribed to be taken occasionally.

13th.—There was little or no pain in the loins, groin, or testicle. The induration and swelling of the testicle and spermatic chord, were much diminished. As the mercurial pills continued to disagree with the bowels, they were discontinued, and a drachm of the following ointment was directed to be applied to the scrotum every night:

℞. Ung. Hydrargyri ℥j.; Camphoræ ʒj. M. fiat unguentum.

17th.—The gums were very sore. The ointment was discontinued.

28th.—Discharged from the hospital, free from disease.

Case IX. *Chronic Inflammation of the Testicles, appearing to depend on Syphilis, and yielding to the mercurial treatment, which relieved the other syphilitic symptoms.*

Luke Pembroke, twenty years of age, admitted into St. George's Hospital, June 10th, 1818. He laboured under inflammation of the iris of the right eye. There was dimness of vision, and there were partial adhesions of the iris to the capsule of the crystalline lens. He was covered with a syphilitic eruption, which in some parts still retained the appearance of clusters of distinct papulæ; while in others, which probably had been clusters of papulæ originally, it had now assumed an appearance nearly the same with that of the venereal lepra. The right testicle was enlarged and indurated. The disease seemed to be in its early stage, as the epididymis was not confounded with the rest of the organ, but could be distinguished from it, forming a separate, hard, knobbed, and irregular tumor. The left testicle was affected in the same manner, but in a somewhat less degree. The testicles were tender on pressure, but not otherwise painful. The patient said that the eruption had appeared about five weeks before he presented himself at the hospital. The affection of the testicles first showed itself about the same period; and the inflammation of the eye took place three weeks afterwards. He gave no account of his having had any primary venereal symptoms, but, from several circumstances, it was evident that no dependance could be placed on this part of his statement.

June 11th.—Blood was taken by cupping from the back of the neck, and a solution of the extract of Belladonna was dropped into the eye.

℞. Calomelanos gr. ij.; Opii gr. ss. fiat pilula bis quotidie sumenda.

14th.—He was directed to rub in ʒj. of the Ung. Hydrargyri every night, continuing the pills.

19th.—Gums very sore. The induration and swelling of the testicles were diminished, and the inflammation of the iris had nearly subsided.

The mercury was now exhibited in more moderate quantity. The testicles soon regained their natural condition.

July 15th.—The eruption had disappeared, leaving slight brown stains and depressions.

August 17th.—The use of mercury was discontinued.

21st.—Discharged as cured.

In the cases which have been just described, the disease was arrested in the early stage, and while the testicle was yet capable of being restored to its healthy condition. But morbid action sooner or later terminates in a morbid alteration of structure; and the following case affords an example of the changes which are produced in the organisation of the testicle, where the disease has been allowed to make further progress.

Case X. *Chronic Inflammation terminating in Disorganisation of both Testicles, and occurring in combination with Disease of the Urethra and Bladder.*

Joseph Heywood, thirty-six years of age, was admitted into St. George's Hospital, on the 16th of March, 1814, labouring under the effects of neglected stricture of the urethra, with symptoms of disease in the bladder, and abscess at the neck of the bladder. He also laboured under disease in the testicles; each of which was enlarged to three or four times its natural size. The swelling was of an oval form, in some parts hard, in other parts elastic. The testicles were tender to the touch, but, at the time of his admission into the hospital, they were otherwise free from pain.

The history which he gave of the disease in his testicles was as follows:—In the beginning of December, 1813, both testicles became swollen and painful. The pain was considerable, but not sufficient to prevent him going about his usual occupations. The swelling gradually increased, and the pain continued, in a greater or less degree, until within a few days of his admission into the hospital.

Some time after his admission, an abscess presented itself in the perineum. The abscess was opened, and a large quantity of pus was discharged. This, however, did not materially relieve the symptoms, and he sunk and died on the 17th of April.

On dissection, the mucous membrane of the bladder was found of a dark colour, from inflammation, and the surface of it was encrusted with lymph. An abscess, of the size of a walnut, was discovered in the substance of the prostate gland. The abscess, which had been opened in the perineum, was very extensive, and communicated with the urethra behind an old cartilaginous stricture.

The testicles had become much reduced in size since the admission of the patient into the hospital: they were firm and hard to the touch. The opposite surfaces of the tunicæ vaginales adhered universally to each other; but the adhesions were of recent date, and easily torn through. In each testicle, among the tubuli testis, were several masses of solid yellow substance, having no distinct organisation, and making about one-third of the bulk of the whole testicle. In some places this newly formed substance was connected to the glandular structure of the testicle by a moderately firm adhesion; in other places it seemed to lie loose in a cavity. The tubuli testis, for the most part, retained their natural appearance: in a few spots, however, they had been converted into a white substance, having the firm consistence, but not the fibrous structure, of ligament.

This species of disorganisation of the testicle is not uncommon. In some instances, no remains of the tubuli testis are perceptible, a white ligamentous substance being every where found surrounding the unorganised yellow tubercle, in place of the glandular structure. The correspondence in the symptoms may be regarded as in itself sufficient to demonstrate the identity of this disease with that in the cure of which mercury may be said to operate as a specific. If, however, any further evidence be required to prove the truth of this opinion, it seems to be afforded by the following case, in which, after the disease had reached its most advanced stage, and had terminated in the formation of the yellow tubercle in one testicle, it began with precisely the same symptoms in the other testicle, and immediately yielded to the exhibition of mercurial remedies.

Case XI. *Chronic Inflammation terminating in the Disorganisation of one Testicle; afterwards affecting the other Testicle, and yielding to the use of Mercury.*

—— Cook, a middle-aged man, admitted into St. George's Hospital, in the winter of 1810, on account of a disease of one testicle. The testicle was enlarged and indurated, and was of an oval shape. At the anterior part there was one spot, which was more prominent than the rest, covered by red skin, and very tender. Believing that these marks indicated the formation of an abscess, and the patient suffering considerable pain, I made a puncture with a lancet: nothing, however, escaped through the puncture, except blood and a small quantity of serum.

The wound made by the lancet became enlarged by ulceration, and the testicle protruded in the form of a fungus. Under these circumstances, (as the disease continued to increase, and the patient's health began to suffer,) it was determined that the testicle should be removed by an operation.

On making a section of the testicle after the operation, the *tubuli* were found in some parts to be still distinct; while in other parts they had the appearance of being converted into a white and somewhat ligamentous substance. In many places, a yellow, solid, unorganised substance, had been deposited among the other structures. A considerable mass of this yellow substance presented itself on the surface of the fungus formed by the protruded testicle, and could be traced nearly into the centre of that organ.*

The wound made by the operation healed readily; but soon afterwards the other testicle became affected in the same way with that which had been removed. There was pain, which was very severe and nearly constant, in the loins, in the course of the spermatic chord, and in the testicle itself, which now became swollen and indurated.

After having for some time employed leeches and other remedies without advantage, I gave the patient mercury internally, so as to affect his general system; and immediately the progress of the disease in the testicle was arrested, the pain and swelling subsided, and he soon left the hospital as cured.

In this case, which occurred sixteen years ago, it was judged proper that the testicle, which had protruded in the form of a fungus, should be removed by an operation; and even at the present day it may, in some cases, be deemed expedient to resort to such an operation, under the same circumstances. These cases are, however, undoubtedly exceptions to a general rule; and, for the most part, not only castration, but even the excision of the fungus, or the destruction of it by escharotics, may be avoided. The following

* The diseased testicle is preserved in Mr. Brodie's collection of preparations of morbid anatomy.

history furnishes an example of the treatment, which I have seldom known to fail in cases of this description.

CASE XII. *Chronic Inflammation of the Testicle, with Protrusion of the Testicle in the form of a Fungus, yielding to the use of Mercury, combined with other treatment.*

—— Arnold, twenty-one years of age, admitted into ST. GEORGE'S HOSPITAL, August 23d, 1816.

Three months before his admission, he experienced a very severe pain, referred to the loins, and followed by inflammation of the right testicle. This had not been preceded by gonorrhœa; nor did he know of any cause to which the disease could be attributed. The testicle became swollen, the swelling being accompanied with a constant burning sensation. At the end of ten weeks, it had attained the size of a man's fist. Leeches were applied, without any benefit. Afterwards an abscess formed, and burst. This was followed by the protrusion of what appeared to be a fungus through the ulcerated opening of the skin. At the time of the patient being admitted into the hospital, this fungus was as large as a small orange, covered by a layer of straw-coloured unorganised substance, adhering closely to its surface. On more careful examination, it was evident that what had the appearance of a fungus was in reality the testicle itself, which had protruded through the ulcer of the integuments, so that only a small part of it actually remained within the scrotum. There was very little pain in the tumor, but some degree of pain in the loins. The patient's general health was unaffected.

August 24th.—He was directed to remain in bed in the horizontal position,* with the scrotum supported by a bandage; and to take ten grains of the Pilula Hydrargyri daily. The surface of the fungus was sprinkled with the powder of the Hydrargyri Nitrico-oxydum, finely levigated; some simple cerate, spread on lint, being laid over the fungus afterwards. This application was repeated daily.

30th.—The gums were somewhat sore. Some part of the straw-coloured unorganised substance had separated, leaving a healthy surface.

September 2d.—The whole of the straw-coloured substance had separated, leaving a red, healthy granulating surface. The skin of the scrotum had contracted over the testicle; the bulk of that part of it which constituted the fungus being, in consequence, a good deal diminished.

24th.—The same treatment had been continued. The fungus

* The maintenance of the horizontal and supine posture, with the scrotum supported by a bandage, appears to be no unimportant part of the treatment in these cases, favouring the retraction of the testicle within the integuments of the scrotum. The *modus operandi* is sufficiently obvious, and corresponds to that by which a long continuance in the same posture will often effect the reduction of an otherwise irreducible hernia.

was now not larger than a large nut; the testicle was felt reduced in size, and covered by the scrotum as usual.

October.—The patient was discharged as cured.

It is not to be supposed, however, that where the disease has proceeded so far as it had in the last instance, that the testicle can ever be, in all respects, restored to its original healthy condition. The state of the testicle afterwards must, of course, depend on the extent to which its organisation has been injured. When it is completely destroyed, the organ continues indurated through the remainder of the patient's life. At first it is larger than natural, but ultimately it becomes much diminished in size. In the winter of 1821, I examined the body of a gentleman, who had for some years laboured under a complicated train of symptoms, which appeared to depend partly on syphilis, and partly on the operation of mercury. In the early stage of his complaints, he had been affected with this disease of one testicle. At the time of his death, the testicle was reduced to the size of a small nutmeg; and, on a section being made of it, there was found in it a mass of solid, yellow, unorganised substance, imbedded in a white, firm, ligamentous substance; and no vestige of the glandular structure was perceptible. It is true that the organ, under such circumstances, can be of no real utility to its possessor; but, on the other hand, the retaining it, for the most part, is productive of no inconvenience; and it enables the patient to avoid the pain and hazard of a serious operation, as well as the disagreeable sentiment which, in many minds, will be connected with the consciousness of having undergone castration.

Although it may not be very important with respect to surgical practice, it would be interesting, as connected with pathological science, to determine in which of the various textures, of which the testicle is composed, a disease, which is thus capable of ultimately causing the destruction of the whole organ, is originally situated. It is evident that it has a more deeply-seated origin than the tunica vaginalis. We might suppose it possible that the fibrous capsule of the tunica albuginea, and the fibrous bands which connect the opposite surfaces of that capsule to each other, and which answer the purpose of supporting the tubuli testis, might be the parts primarily affected, if we did not find the disease sometimes to begin in the epididymis, in which the tunica albuginea, and the fibrous bands which have been alluded to, are alike wanting. The obvious conclusion is, that the morbid action commences in the glandular structure of the testicle; and this opinion is confirmed by the following case and dissection.

Case XIII. *Chronic Inflammation of the Testicle, terminating in the Disorganisation of the Testicle, and the Protrusion of it in the form of a Fungus; with the Appearances observed on the Dissection of the diseased Organ.*

John Smith, twenty-three years of age, admitted into St. George's Hospital, March 24th, 1819.

Three months ago he fell from a tree, and hurt his left testicle. He had some pain, which subsided in a few minutes. He continued well for three weeks, when he experienced a slight pain in the testicle, which gradually became more severe. About a week after the pain began, a swelling took place at the upper and anterior part, which gradually extended to the whole testicle. In the course of a few weeks an abscess formed, and burst on the anterior part of the scrotum, discharging a small quantity of matter.

At the time of his admission into the hospital, the left testicle was swollen to about four times its natural size. The swelling was hard, and nearly globular. Where the skin had ulcerated, there was a sore as large as a shilling, with an imperfect granulating surface. The spermatic chord at the posterior part, in the situation of the vas deferens, was slightly indurated. There was an occasional pricking pain in the testicle, and also a dull pain in the course of the spermatic chord. He had lost flesh, his countenance was sallow, and his digestion was impaired. He was directed to take the Decoction of Sarsaparilla; and, some days afterwards, the Pilula Hydrargyri was combined with it.

The mercury disagreed with his bowels, so that it was taken very irregularly. His general health became more impaired; the ulceration of the scrotum became more extensive, and the testicle protruded through the ulcerated opening, having the appearance of an ill-conditioned fungus. Under these circumstances, it was thought advisable to remove the testicle by an operation.

On dissecting the testicle which had been removed, it was found to be of four or five times its natural size, and of an irregular shape. That which appeared like a fungus was the glandular structure of the testicle, the membranes covering it being in a state of ulceration. On a section being made of the testicle, it was found to contain a considerable quantity of solid, yellow, unorganised substance, similar to that which was observed in the former cases, but not collected in large masses; and in the intermediate spaces there was common coagulated lymph, in which some remains of the tubuli testis were observable. The vas deferens, where it terminated in the epididymis, externally had a natural appearance; but, on slitting it open longitudinally, a yellow substance was found adhering to its inner surface, apparently corresponding to that which had been observed in the testicle itself; and the membrane lining the vas deferens, after the yellow substance had been removed, was thickened, and apparently preternaturally vascular. This led me to make a similar examination of the tube of the epididymis, by slitting it open in the same manner, as far as its size was sufficient to admit of this being done. The appearances corresponded to those observed in the lower part of the vas deferens; the inner membrane being thickened, with a layer of solid yellow substance adhering to it.*

It has been seen that, in some of the cases which have been related, a small quantity of serous fluid was found to have been effused into the cavity of the tunica vaginalis. Now, this collection of fluid occasionally amounts to several ounces, and the disease of the testicle thus becomes complicated with a considerable hydrocele. Under these circumstances, the most prudent plan is to subject the patient, in the first instance, to the influence of mercury; and, if the hydrocele should not disappear spontaneously after the affection of the testicle is cured, it may be treated in the usual manner, by the injection of a stimulating liquid into the tunica vaginalis. But we may sometimes fail in making an exact diagnosis, since the formation of hydrocele is not unfrequently attended with some degree of pain in the first instance; and the thickening of the tunics by which the testicle is immediately invested, which is not very uncommon in hydrocele, and which forms no objection to the operation, gives to the fingers an impression very similar to that which is perceived in cases of chronic inflammation of the glandular structure. The following case shows what may be the effect of injecting a hydrocele, where this complication exists; and it also shows how the ill consequences of the injection may be obviated.

CASE XIV. *Chronic Inflammation of the Testicle combined with Hydrocele, in which a succession of Abscesses followed the Operation of Injection, and the disease was ultimately cured by the use of Mercury.*

In April 1817, I was consulted, in conjunction with Mr. FERNANDEZ, by a gentleman from Barbadoes, respecting what appeared to be a hydrocele. Several ounces of fluid were drawn off by means of a trocar, and the tunica vaginalis was injected with equal parts of port-wine and water. When the tunica vaginalis was emptied of fluid, the testicle was felt harder and larger than natural. The inflammation induced by the injection did not subside as usual. An abscess formed, with a moderate degree of pain, which was punctured about three weeks after the operation, and which discharged several ounces of pus. This abscess continued to discharge matter for several days, and then began to heal;

* In this case, that which appeared to be a fungus was, in fact, the glandular structure of the testicle, which had protruded through an ulcerated opening of the scrotum. There may, however, be fungous tumors of the testicle formed in a different manner, and depending on other causes. My friend Professor MACARTNEY, of Trinity College, Dublin, informs me that he has a preparation, in which the glandular structure of the testicle is perfectly free from disease, while a fungus has arisen from the external surface of the tunica albuginea.

but the bulk of the tumor did not diminish. Several abscesses formed in succession; and, at the end of between two and three months from the time of the operation, the testicle was still a large indurated mass, with abscesses opening on its surface, and discharging matter. Mercury was now administered so as to affect the gums; and, immediately on the gums becoming sore, the testicle began to diminish in bulk, the abscesses healed, and in less than a month the patient was quite well, except that the left testicle was somewhat harder and larger than natural.

Although there are so many cases of chronic inflammation of the testicle in which mercury is useful, there are other forms of the disease in which it is productive of no benefit, or absolutely injurious; or chronic inflammation of the testicle may even be the consequence of the use of mercury acting on a particular constitution, and in this case Sarsaparilla is likely to afford the most effectual means of cure.

Case XV. *Chronic Inflammation of the Testicle with Abscess, yielding to the use of Sarsaparilla.*

—— Jarvis, twenty-seven years of age, admitted into St. George's Hospital, September 16th, 1812.

He had a painful tumor, formed by thickening of the periosteum of the frontal bone; and pains in his shoulders and legs, which were aggravated in the night when warm in bed. The right testicle was enlarged, and slightly indurated; and there had been in it an abscess, which had burst, and left a sore with a margin of red skin. There was little or no pain in the testicle.

He said that, two years before his admission into the hospital, he had a chancre, for which he took mercury internally, going about his occupations as usual. He continued to take mercury for two or three months. Two months after the mercury was left off, he became affected with the pains in his limbs and forehead. He had no sore of the throat, nor eruptions, nor any other symptoms.

He was directed to take Decoction of Sarsaparilla Oj., Ext. of Sarsaparilla ʒij., daily.

When he had taken this medicine for about a week, the pains had nearly left him, and the tumor in the forehead was much diminished.

October 17th.—The tumor in the forehead had nearly disappeared, and he did not complain of pain. The testicle was smaller, and the discharge from the abscess was diminished.

November 1st.—The abscess of the testicle was healed: he was well in all other respects. Discharged as cured.

In this instance, the character of the diseased testicle was such as might have led me to believe that the disease itself was different from that which existed in the cases before described. However, the result of my experience, on the whole, is that our diagnosis, in such cases, ought to be founded less on the present symptoms, than on the past history; and not so much on the history of the symptoms, as on that of the remedies which have been employed. If chronic inflammation of the testicle begins during a course of mercury, and be combined with such symptoms as mercury is likely to have produced, it is, at any rate, prudent not to administer mercury in the first instance; and the probability is, that a proper exhibition of sarsaparilla will be adequate to the cure.*

* It is not in our power to insert the whole of this communication in the present Number of our Journal. The remaining part of it will be published in the Number for November.—ED.

